# The Healing Environment of Dental Clinics through the Eyes of Patients and Healthcare Professionals: A Pilot Study

**DOI:** 10.3390/ijerph192013516

**Published:** 2022-10-19

**Authors:** Maria Sarapultseva, Alena Zolotareva, Natal’ya Nasretdinova, Alexey Sarapultsev

**Affiliations:** 1 Department of Pediatric Dentistry, Medical Firm Vital EBB, 620144 Ekaterinburg, Russia; 2 School of Psychology, HSE University, 101000 Moscow, Russia; 3 Autonomous Non-Commercial Organization «Association Stomatology», 620102 Ekaterinburg, Russia; 4 Russian-Chinese Education and Research Center of System Pathology, South Ural State University, 454080 Chelyabinsk, Russia; 5 Ural Division of Russian Academy of Sciences, Institute of Immunology and Physiology (IIP), 620002 Ekaterinburg, Russia

**Keywords:** ASPECT, dental clinic, dentistry, healing environment, work environment

## Abstract

The physical environment of healthcare settings can promote both the healing process and patient feelings of well-being, as well as instill positive emotions in employees. The present study aimed to evaluate the dental work environment of a typical private and public dental clinic to identify key parameters that determine the perception of health facilities by patients and employees. The study was carried out from 1 to 20 December 2021, in two dental clinics in Ekaterinburg (Russian Federation) using ‘ASPECT’. The participants were 58 staff and 94 patients. The results showed that, compared with patients, staff reported higher views scores, nature and outdoors scores, and comfort and control scores. The common criterion that distinguishes private clinics from public ones was comfort and control. Compared with patients in state clinics, patients in private clinics reported higher privacy, company and dignity scores, comfort and control scores, interior appearance scores, and facility scores. In general, while views scores and nature parameters can be singled out as a universal absolute value for everyone in a particular environment, staff pay more attention to factors that contribute to long-term comfortable stay and performance of their duties.

## 1. Introduction

The place at which patients and physicians meet plays a special role in the interaction between patients and doctors, that is, the medical clinic or the hospital (dental office in the case of dentistry). Today, healthcare facilities are considered among the most complex institutional structures, not only in terms of complicated medical supplies, but also in terms of some sensitive problems, such as the psychological needs of users [1,2,3].

Taking into account medical centers as part of the healing environment, which might affect the well-being of people there, researchers focus their studies almost entirely on visitors or patients, but employees spend as much, if not more, time at these places.

Oral diseases are one of the most common chronic diseases and are a public health concern owing to their prevalence, treatment costs, and impact on individuals and society [4]. Although there is no universal agreement on how often people should see a dentist, several countries recommend that children visit a dentist at least once a year to prevent and cure problems as quickly as possible, while adults without problems can wait up to two years. On average, in EU countries, people have only one consultation with a dentist in a year [5], spending about an hour in the clinic. Furthermore, the available data from the FDI and WHO suggest that there are at least 1.6 million dentists worldwide, unevenly distributed in the six WHO regions [6] (these estimates do not include junior medical and auxiliary personnel). According to the forecasts, the annual number of dental school graduates, as well as the number of dentists per capita, will increase in the next few years [7].

The physical environment of healthcare settings can promote both the healing process and patient feelings of well-being [8,9], as well as instill positive emotions in employees, increasing their passion and overall satisfaction [10,11,12,13]. Furthermore, studies have suggested that the design, visibility, and accessibility levels of healthcare settings can improve patient care and treatment results by reducing medical errors and waste and improving the level of communication and teamwork [14,15]. According to the literature, thermal comfort, acoustic comfort, visual comfort, and indoor air quality are the main parameters that determine indoor environmental quality in buildings, reviewed in [3,13,16]. Indoor air quality and thermal comfort in a hospital environment can improve staff productivity, while, for the patient, it leads to a reduction in stay duration and accelerates the healing process [16]. Sound quality is a particular factor that has an influence on both the patient’s healing process and the output of hospital staff. A noisy and unpleasant hospital environment can increase patient worry, cause respiratory problems and stress development [16], and even affect job satisfaction [12,17,18,19]. Despite the significant number of studies conducted in hospital facilities, studies conducted in specialized medical institutions (such as primary care centers, dental clinics, dialysis centers, plastic surgery, or cosmetology) are quite rare [20,21]. Furthermore, studies conducted in dental clinics are mainly aimed at assessing environmental perception and the factors that determine it through the eyes of clinic patients [22,23,24,25,26,27,28]. Most of them attempted to determine patient preferences regarding dental waiting area and operatories [23,25,27,28] and to assess the impact of aspects supporting sensory conditions (colors, light, and spatial organization); reassurance strategies (decorations, dental team attire, and drawings); and anxiety control strategies (play area, TV, and toys) [29,30]. The majority of patients liked open windows, a wall covered with pictures, living plants, music, and the freedom for children to play in the waiting area [23,24,25].

In general, even minor changes made to the design of health facilities (waiting rooms, operating rooms) have been shown to have a significant effect on how a person perceives the coming dental experience [23]. However, evaluations of dental workplace environments have been based primarily on the physical workload or have been based primarily on a pathogenic perspective, that is, with an emphasis on disease and risk factors [31,32,33,34,35]. We found no data on the perception of the design and environment of dental clinics by employees and staff, nor any data on how the evaluation of the same institutions by patients and medical professionals correlates. Therefore, the present study aimed to evaluate the dental work environment of typical private and public hospitals to identify key parameters that determine the perception of health facilities by patients and employees. The null hypothesis was that there would be no differences in key parameters that affect the perception of medical facilities by patients and employees, regardless of their role (patient or employee) and type of clinic (public or private). The alternative hypothesis was that differences in key parameters that affect the perception of healthcare facilities by patients and staff could depend on their role or type of clinic.

## 2. Materials and Methods

### 2.1. Study Design

This is a cross-sectional study using survey methodology and convenience sampling involving 100 adult patients and 60 staff from two dental clinics (private clinic and government center) in Ekaterinburg, Russia. The study was carried out between 1 December and 20 December 2021. To be eligible for participation, the respondents had to meet the following inclusion and exclusion criteria. Inclusion criteria included the following: (1) working in a dental clinic during the study, defined as the period from 1 January 2021 to 30 December 2021; (2) using dental services in the aforementioned clinics during the study; and (3) providing informed consent to participate in the study by replying Yes. There was no target recruitment size. Direct comparisons were not drawn; therefore, no power calculation was performed.

The study proposal and protocol approved by the Ethics Commission of Chelyabinsk State University (17 November 2020) as part of the joint project “Russian-language adaptation of diagnostic scales to assess psychological conditions caused by various stressful and traumatic life events”.

**Figure 1:**
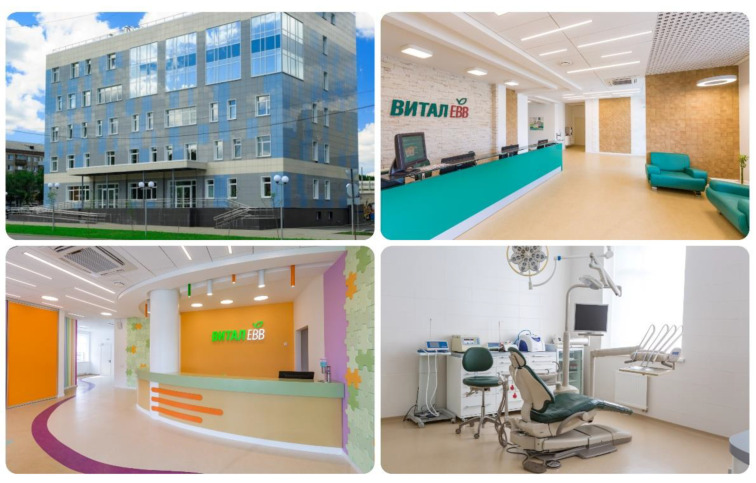
Environment of the private dental clinic.

**Figure 2:**
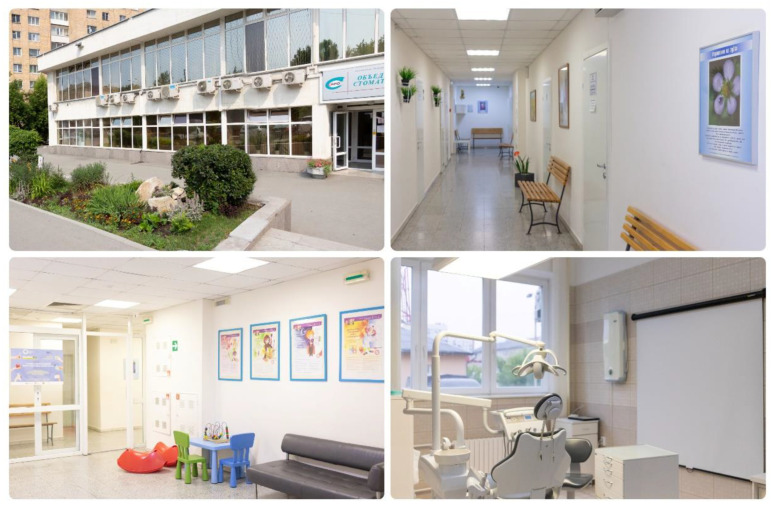
Environment of the government dental clinic.

The data were collected through a self-administered questionnaire. The quality of the dental clinic’s healing environment (Figure 1 and Figure 2) was evaluated using some of the established dimensions of ‘ASPECT’. ASPECT is a tool to evaluate the quality of design of patient and staff environments in healthcare facilities [36,37]. It presents a profile that indicates the strengths and weaknesses of a design or an existing building and can be used as a standalone form or for evaluation workshops. Each indicator can be weighted as high (2), normal (1), or zero (0) and is evaluated with a six-point Likert scale. The evaluated indicators include privacy, compatibility, and dignity (patients’ privacy and dignity must be maintained while in health facilities); views, nature, and outdoor (the degree to which patients can see outside and around the building); comfort and control (hospital layout should minimize unwanted noise in patient areas and patients should also be able to easily control internal temperature and lighting); legibility of the place (building layouts should be clear and easy to understand, so patients can easily find their way with ease); and interior appearance (patient spaces should feel homely, while interior spaces should feel light and airy; have a variety of colors; and look clean, tidy, and cared for) [38]. All of these factors influence the satisfaction of patients with the overall delivery of health care.

### 2.2. Statistical and Data Analysis

Firstly, preliminary analyses consisted of calculating the frequencies and percentages for categorical variables and means and standard deviations for numerical variables. Secondly, Student’s *t*-test and ANOVA were used to compare means between various groups of participants. A *p*-value of <0.05 was considered statistically significant.

## 3. Results

The study involved 160 volunteers, including 60 staff and 100 patients. After excluding questionnaires with missing values from the analysis, the final sample consisted of 152 respondents, including 58 staff and 94 patients. The descriptive characteristics of the participants are presented in Table 1.

**Figure 3:**
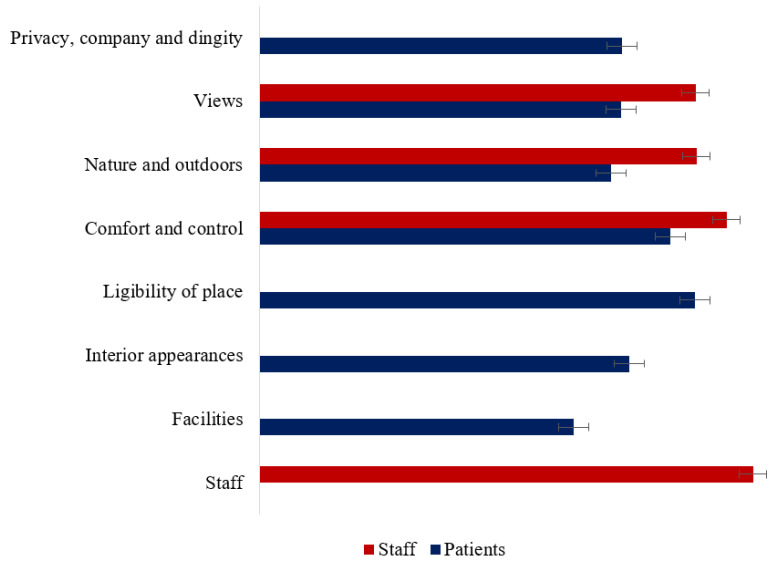
Healing environment for staff and patients.

Figure 3 illustrates the healing environment scores for staff and patients. Compared with patients, staff reported higher views scores (t = 4.522, *p* < 0.001, d = 0.656), nature and outdoors scores (t = 4.979, *p* < 0.001, d = 0.770), and comfort and control scores (t = 3.189, *p* = 0.002, d = 0.532).

### 3.1. Healing Environment for Staff

Sex differences were not tested owing to the small number of males. There were no statistically significant correlations between staff age and healing environment scores (all *p*-values > 0.05) and between experience and healing environment scores (all *p*-values > 0.05)

Private clinic staff reported higher comfort and control scores (t = 3.254, *p* = 0.002, d = 0.880) and staff scores (t = 2.593, *p* = 0.012, d = 0.702) than state clinic staff. No statistically significant differences were found between the estimates of state and private clinic staff for views scores (t = 1.731, *p* = 0.089, d = 0.469) and nature and outdoors scores (t = 1.704, *p* = 0.094, d = 0.461).

Dental auxiliaries reported higher staff scores than dentists and dental assistants (F (2,55) = 3.751, *p* = 0.030, η2 = 0.120). No statistically significant differences were found between dentists, dentist assistants, and dental auxiliaries for view scores (F (2,55) = 0.477, *p* = 0.623, η2 = 0.017), nature and outdoors scores (F (2,55) = 0.362, *p* = 0.698, η2 = 0.013), and comfort and control scores (F (2,55) = 0.644, *p* = 0.529, η2 = 0.023).

### 3.2. Healing Environment for Patients

Females reported higher views scores than males (t = 2.048, *p* = 0.046, d = 0.492). No statistically significant differences were found between women and men for privacy, company, and dignity scores (t = 0.028, *p* = 0.978, d = 0.006); nature and outdoor scores (t = 1.046, *p* = 0.298, d = 0.225); comfort and control scores (t = 1.753, *p* = 0.083, d = 0.376); place legibility scores (t = 0.188, *p* = 0.852, d = 0.036); interior appearance scores (t = 1.025, *p* = 0.038, d = 0.220); and facilities scores (t = 0.807, *p* = 0.422, d = 0.173). There were also no statistically significant correlations between patient age and healing environment scores (all *p*-values > 0.05).

Compared with patients in the state clinic, patients in the private clinic reported higher privacy, company, and dignity scores (t = 5.910, *p* < 0.001, d = 1.199); comfort and control scores (t = 5.578, *p* < 0.001, d = 1.083); placeability scores (t = 6.119, *p* < 0.001, d = 1.204); interior appearance scores (t = 5.176, *p* < 0.001, d = 1.056); and facilities scores (t = 7.318, *p* < 0.001, d = 1.481). No statistically significant differences were found between patients in state and private clinics for views scores (t = 0.933, *p* = 0.353, d = 0.193) and nature and outdoors scores (t = 1.950, *p* = 0.054, d = 0.404).

## 4. Discussion

There are three different types of people who occupy hospitals: patients, staff, and visitors. For staff, the hospital serves as their permanent workspace; for patients, it serves as a temporary residence where environmental conditions can be secondary because the patient’s main concern is recovering from their illness [39]. Although the objective of the study was to evaluate the perception of the clinic environment by both patients and staff, we did not select visitors as a separate group, as, given the specifics of dental appointments (relatively short patient stays and duration of procedures, lack of inpatient treatment), dental clinics generally do not have visitors visiting patients.

The null hypothesis was that there were no differences in key parameters that affect the perception of medical facilities by patients and employees, regardless of their role or type of clinic. However, the results of the present study showed several significant differences in the perception of healthcare facilities between patients and dentists. This finding broadly supports the work of other studies in this area that have reported different needs for things and patients (visitors) [13]. According to the results, compared with patients, staff reported higher view scores, nature and outdoors scores, and comfort and control scores. Given the considerable amount of time staff spend in healthcare facilities, it seems reasonable that the nature of their environment influences how they feel and perform. Interestingly, nature and the environment are per se influential, regardless of the type of clinic (private or state). An indirect proof of the latter is that no statistically significant differences were found for views scores and nature and outdoors scores between staff in state and private clinics or between dentists, dentist assistants, and dental auxiliaries. This result may be explained by the fact that, according to Sebba (1991), the places adults remember the most in childhood indicated that the outdoors is the most important environment for 95.6% of men and women [39].

At the same time, it is extremely important that the great importance of nature in the eyes of people is accompanied by its beneficial effect on them [40,41,42,43]. Several studies have shown that increased green space availability is consistently associated with increased perceived restoration [23,24,25]. Furthermore, the results of physiological and verbal measures converged to indicate that subjects exposed to natural and non-urban environments recovered more quickly and completely, and the pattern of physiological findings suggested that responses to nature include a prominent component of the parasympathetic nervous system [43]. Today, restorative influences of nature are considered to involve a shift towards a more positive emotional state, positive changes in physiological activity levels, and a reduction in stress [44].

Differences in patient and staff perceptions of state and private clinics were also revealed. Private clinic staff reported higher comfort and control scores and staff scores than state clinic staff. Patients in private clinics reported higher privacy, company, and dignity; comfort and control scores; legibility of place scores; interior appearance scores; and facility scores than those in state clinics. According to the survey results, the common criterion distinguishing private clinics from public was comfort and control, which may be because of less bureaucracy and hierarchy in private clinics. Health care organizations have a reputation for being rigid and difficult to manage [45], and centralization, bureaucracy, and severe dependence on government with the strong hierarchical structure of state hospitals can strongly affect staff perception and possibly work productivity [46]. According to the literature, the sector of employment (private or public) has a significant association with the prevalence of stress and depression, and workers in the public sector are less likely than their counterparts in the private sector to feel supported when they disclose mental health problems [47]. However, these associations can be determined by the character of the health system in each individual country and the relative importance of the measured aspects can differ between cultures [48]. For example, in India, private sector employees were found to be more depressed than public sector employees and the emotional well-being of dental professionals working in the government sector was significantly better than that of those working in the private sector [49,50].

Gender differences in patient perception were also revealed. Women reported higher views scores than men. This finding broadly supports the work of other studies in this area that highlight that the importance placed on environmental aspects is perceived more widely by women [51,52]. Prior surveys such as those conducted by Mourshed and Zhao (2012) have shown that female healthcare providers are more perceptive to factors related to sensory environments, such as visual, acoustic, and olfactory factors, as compared with their male counterparts [53].

## 5. Conclusions

The results of this study highlight differences in the perception of the environment by patients and staff in the dental clinic. While views scores and nature parameters can be singled out as a universal absolute value for everyone in a particular environment, staff pay more attention to factors that contribute to long-term comfortable stay and performance of their duties (the parameters of comfort and control).

The comparative analysis of the two types of clinics also revealed the potential importance of comfort factors. According to Ulrich’s (1991) theory of supportive design, the hospital environment will reduce stress if it fosters perceptions of control, social support, and positive distraction [43,54,55,56]. The results obtained allow us to draw two conclusions. First, based on the evaluations given by visitors and employees of the clinics studied, one can assume that private clinics are more consistent with the postulates of Ulrich’s (1991) theory of supportive design [43,54,55,56]. Even more importantly, there is evidence that people do not have conflicts between their preferences for the main parameters of the premises and the usefulness of the latter for their health and well-being.

In general, despite the limitations of this study, the insights gained from it can help architects and healthcare managers find the characteristics of clinic design that can offer the co-benefits of promoting health and comfortable working conditions [57].

As the present study confirmed data from other studies that defined views and nature parameters as universal key characteristics to which people react regardless of their role (patient or doctor), it is planned to change the intensity and quality of these parameters (via planting greenery in clinic areas and view panels) in order to evaluate their impact on the perception of the clinic and the level of concern of patients through additional questioning using reliable and valid scales to assess psychological comfort and distress. In the future, for some cohorts of patients (who are being treated under anesthesia and, as a result, under instrumental monitoring), it is planned to simultaneously record such parameters as heart rate and cortisol levels in saliva, which will make it possible to instrumentally assess the severity of stressful psychogenic load. With the expansion of work to general practice hospitals, it is possible to determine a larger number of parameters and a more subtle analysis of the parameters and terms of recovery.

Moreover, large-scale future studies will allow us not only to determine the most important parameters that affect the assessment of the environment by people, and rank them according to their importance, but also to integrate them with data on the influence of environmental parameters on well-being and working capacity. As a result, an integrated system should be obtained to assess and predict the impact of the environment on people. In addition, more research could usefully explore whether different groups of hospital patients could have different perceptions of indoor environmental quality.

## 6. Limitations of the Study

Owing to the pilot nature, the generalizability of the study’s results is subject to certain limitations. First, the study was subject to selection bias and sampling error, because all data were obtained from patients and cohorts of staff admitted to only two dental clinics in Ekaterinburg, Russia. The sample of participants was not representative and, therefore, the study was more of a pilot type. Selection bias and response bias may have resulted in an overestimation or underestimation of the environmental impact. Furthermore, human–environment interactions are culturally bound [56]; therefore, more cross-border collaborations and research are needed. The chosen methodology did not allow us to study the relationship between the healing environment and job satisfaction of employees, as well as the perceived quality of medical care by patients. Finally, the study was not designed or intended to demonstrate an effect on mental health and productive work of staff or treatment outcomes (the final results of evidence-based healthcare design) for patients.

## Figures and Tables

**Table 1:** Participants and descriptive characteristics.

	Staff (*n* = 58)	Patients (*n* = 94)
Age, mean (SD)	39.57 (9.15)	34.61 (8.59)
Sex		
Male, *n* (%)	8 (13.8)	34 (36.2)
Female, *n* (%)	50 (86.2)	60 (63.8)
Clinic		
Private, *n* (%)	36 (62.1)	43 (45.7)
State, *n* (%)	22 (37.9)	51 (54.3)
Experience, mean (SD)	16.77 (8.92)	n/a
Work position		
Dentist, *n* (%)	18 (31)	n/a
Dentist assistant, *n* (%)	12 (20.7)	n/a
Dental auxiliaries, *n* (%)	28 (48.3)	n/a
Healing environment		
Privacy, company, and dignity, mean (SD)	n/a	3.72 (1.37)
Views, mean (SD)	4.47 (0.70)	3.71 (1.36)
Nature and outdoors, mean (SD)	4.48 (0.91)	3.60 (1.27)
Comfort and control, mean (SD)	4.79 (0.88)	4.21 (1.21)
Legibility of place, mean (SD)	n/a	4.46 (1.10)
Interior appearances, mean (SD)	n/a	3.79 (1.16)
Facilities, mean (SD)	n/a	3.22 (1.16)
Staff, mean (SD)	5.06 (0.98)	n/a

Note. n/a = not applicable.

## Data Availability

The datasets analyzed during the current study are available from the corresponding author upon reasonable request as they contain information on the gender, age, work experience, and places of work of the respondents.
